# Early Postoperative Adverse Events (≤ 30 Days) in Spinal Oncology: Defining the Incidence, Severity, and Predictors in a Mixed Prospective Cohort Including Intradural and Metastatic Spinal Tumors

**DOI:** 10.1002/ijc.70640

**Published:** 2026-07-09

**Authors:** Meltem Ivren, Philip Dao Trong, Maximilian Klass, Martin Dugas, Sebastian Ille, Sandro M. Krieg, Pavlina Lenga

**Affiliations:** ^1^ Department of Neurosurgery Heidelberg University Hospital Heidelberg Germany; ^2^ Medical Faculty of Heidelberg University Heidelberg Germany; ^3^ Vivantes Klinikum Neukölln Vivantes Netzwerk für Gesundheit GmbH Berlin Germany; ^4^ Institute of Medical Informatics Heidelberg University Hospital, Heidelberg University Heidelberg Germany

**Keywords:** adverse events, Clavien–Dindo, comorbidity, risk stratification, spinal tumors

## Abstract

Complication reporting in spinal tumor surgery remains inconsistently defined. We aimed to quantify the incidence, severity, and spectrum of early (≤ 30 days) postoperative adverse events (AEs) in spinal tumor surgery and to identify predictors of overall and serious complications using standardized severity grading. We analyzed prospective data from 156 patients undergoing spinal tumor surgery for mixed primary and metastatic pathologies between 2023 and 2025. Intra‐ and extradural lesions were included. AEs were classified according to the Clavien–Dindo system, and risk factors were identified using logistic regression. The age‐adjusted Charlson comorbidity index (ACCI) was used to assess comorbidity burden. Any postoperative AE occurred in 16.7% of cases, and serious complications (Clavien–Dindo ≥ III) occurred in 7.1%. Surgery‐related events predominated (14.7%). Stratified analysis revealed differences between tumor subgroups, with the highest complication rates observed in intramedullary tumors, primarily driven by neurological deficits. The ACCI was the strongest predictor of both overall (*p* = 0.006) and serious complications (*p* = 0.001), corresponding to a 20%–39% risk increase per point. Emergency surgery and advanced age were associated with serious complications, while operative duration correlated modestly with overall AEs. Mortality within 30 days was 1.9%. AEs were dominated by neurological and surgery‐related complications. Complication profiles differed according to tumor location, reflecting underlying surgical and biological differences. Comorbidity burden and urgency emerged as predictors for AEs in general. These findings underscore the need for structured preoperative risk stratification, timely elective referral, and standardized AE reporting to improve safety and comparability across spinal tumor surgery studies.

## What's New?

High‐quality data on the incidence and severity of complications following spinal tumor surgery are scarce and inconsistent. This prospective single‐center study provides a contemporary analysis of early postoperative adverse events after spinal tumor surgery using standardized Clavien–Dindo grading. The results showed a moderate overall complication burden but a clinically meaningful rate of serious adverse events and reoperations. Both the comorbidity burden, quantified by the age‐adjusted Charlson Comorbidity Index, and emergency surgery were significant predictors of severe postoperative complications. These findings underscore the importance of individualized perioperative risk stratification beyond tumor histology and the need for standardized adverse events reporting.

## Graphical abstract

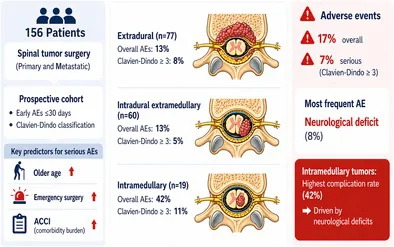

**Abbreviations**
ACCI: age‐adjusted Charlson‐comorbidity index
AE: adverse event
CSF: cerebrospinal fluid
IDEM: intradural extramedullary
NSQIP: National Surgical Quality Improvement Program
POPAE: Postoperative Adverse Event
SAVES: Spine Adverse Events Severity System
SAVES‐V2: Spine Adverse Events Severity System, Version 2

## 1 Introduction

Spinal tumors represent a diverse and increasingly prevalent subset of neurosurgical pathology. Population‐based analyses indicate that the incidence of certain primary subtypes—such as spinal schwannomas [1] and ependymomas [2]—is rising over time, paralleled by a marked increase in spinal metastatic involvement [3]. Collectively, these trends underscore a growing clinical and socioeconomic burden, as spinal neoplasms often present with pain, neurological deficits, and mechanical instability that require urgent or complex surgical intervention.

Despite major advances in surgical technique, imaging, and perioperative care, high‐quality data on complication incidence and severity remain scarce and inconsistent. Reporting standards in spinal tumor surgery are highly heterogeneous: a systematic review across major spine journals found that many studies did not report complications at all, and when they did, divergent classification systems and follow‐up definitions were used [4]. This variability hampers interstudy comparison, complicates quality benchmarking, and limits the development of evidence‐based risk models.

To address these limitations, standardized adverse event (AE) frameworks have gained prominence. Spine‐specific tools such as the Spine Adverse Events Severity System (SAVES) and its updated SAVES‐V2 version have demonstrated strong reliability and interobserver consistency for AE capture in both elective and oncologic spine surgery [5]. In parallel, the Clavien–Dindo classification, originally developed for general surgery, has been validated in neurosurgical practice and provides a universal, therapy‐oriented measure of complication severity [6]. The combined use of these frameworks enables granular and reproducible assessment of both event incidence and clinical impact.

Given the increasing complexity of spinal tumor care and the absence of unified complication benchmarks, a better understanding of who develops AEs and why is clinically crucial. Such insight informs patient counseling, supports resource allocation, and highlights modifiable risk factors—particularly in frail or high‐acuity populations where morbidity directly affects neurological recovery and survival.

Against this background, the present study aimed to (i) quantify the incidence and spectrum of AEs following spinal tumor surgery within the early postoperative period (≤ 30 days) using a standardized severity framework and (ii) identify independent patient‐ and surgery‐related predictors of overall and serious complications, thereby contributing to improved perioperative risk stratification and safety in spinal oncology.

## 2 Methods

### 2.1 Study Design

This study analyzes data collected between January 2023 and January 2025 from a prospectively compiled database in a single tertiary center. All adult patients with histologically confirmed spinal tumors (regardless of malignancy) undergoing surgery at our center during the specified study period were included. Pediatric patients were excluded. We assessed data about patient demographics and comorbidities, surgical strategy, tumor histology and postoperative adverse events (POPAE) in order to evaluate the incidence and predictors of postoperative complications in spinal tumor surgery.

### 2.2 Assessment of POPAE

Within the standard clinical routine of our institution, a POPAE form is completed for every patient across all neurosurgical subspecialties at discharge from our department, including cases of death or referral to other units. The forms are first filled out by the respective resident neurosurgeon responsible for the ward—then reviewed by a responsible senior attending. Hence, roughly 15 senior attendings and 15 resident neurosurgeons actively feed and monitor the database [7, 8]. Once reviewed, the data is added to a continuously growing POPAE database [7]. All POPAE forms are quarterly reviewed. The neurosurgical team of each ward is notified when there is a resubmission of a patient within 30 days [7]. The POPAE database is regularly crosschecked with the hospital administration database [8]. Complications analyzed in this study were restricted to those occurring within 30 days postoperatively, in line with commonly used early outcome definitions [9, 10, 11]. Long‐term AEs beyond this period were not included as described in previous study protocols [7, 8].

### 2.3 Categorization of AEs

Categories of surgical complications included impaired wound healing, CSF (cerebrospinal fluid) fistulas, CSF infection, malpositioned instrumentation, postoperative hemorrhage and new neurological deficits. Non‐surgical complications included events not directly related to surgery such as urinary tract infections or cardiac events. Postoperative AEs were classified according to the Clavien‐Dindo classification system: Grade I: minor deviations from the normal postoperative course not requiring pharmacological or procedural intervention; Grade II: complications require pharmacological treatment; Grade III: complications necessitate surgical, endoscopic, or radiological intervention; Grade IV: complications are life‐threatening and require intensive care management; Grade V: death [12].

### 2.4 Demographic and Clinical Variables

We assessed age, sex, the ACCI [13, 14], tumor histology and localization. Surgical parameters included duration of surgery, estimated blood loss, urgency of surgery (emergency vs. elective surgery), and surgical strategy. Surgeries were considered elective when scheduled ≥ 1 day in advance. Data completeness was high, reflecting the prospective and standardized data collection, with missing values in only 2 cases (1.3%) for duration of surgery; all other variables were complete.

### 2.5 Statistical Analysis

Data analysis was performed with SPSS (IBM SPSS Statistics version 30, IBM corp.). Normality was tested using the Shapiro–Wilk test. Variables are listed as numbers and percentages (categorical variables) or as means ± standard deviation (continuous variables). We performed a logistic regression analysis to identify predictors for complications overall as well as serious complications (Clavien‐Dindo ≥ 3). A significance level of *p* ≤ 0.05 was applied.

## 3 Results

### 3.1 Overall Cohort

Overall, 156 patients with a median age of 59 years (range 21–86) were included in this study. Gender distribution was almost equal (female = 82, 52.6%). The most common histologies were metastases (*n* = 53, 34.0%), schwannomas (*n* = 32, 20.5%), meningiomas (*n* = 25, 16.0%) and ependymomas (*n* = 22, 14.1%). Within the metastases subgroup, the most common primaries were lung (*n* = 14, 26.4%), prostate (*n* = 10, 18.9%) and breast (*n* = 6, 11.3%). The mean ACCI was 2. Stratified analysis by tumor location revealed distinct subgroup characteristics: Extradural tumors (*n* = 77) were predominantly metastatic and associated with a higher comorbidity burden (median ACCI 3), whereas intradural extramedullary tumors (*n* = 60) were mainly composed of benign entities such as meningiomas and schwannomas (median ACCI 1). Intramedullary tumors (*n* = 19) were characterized by intrinsic spinal cord pathologies, particularly ependymomas and gliomas. The demographic parameters are summarized in Table 1.

**TABLE 1:** Baseline characteristics.

Localization	Overallcohort	Extradural	Intraduralextramedullary	Intradural intramedullary
	*n* = 156	*n* = 77	*n* = 60	*n* = 19
Age (median, range)	59 (21–86)	62 (21–86)	55 (27–85)	60 (27–83)
Sex				
Female (*n*, %)	82 (52.6)	33 (42.9)	41 (68.3)	8 (42.1)
Histology (*n*, %)				
Metastasis	53 (34.0)	47 (61.0)	3 (5.0)	3 (15.8)
Lung	14 (9.0)	14 (18.2)	0 (0.0)	0 (0.0)
Prostate	10 (6.4)	9 (11.7)	1 (1.7)	0 (0.0)
Breast	6 (3.8)	5 (6.5)	0 (0.0)	1 (5.3)
Kidney	5 (3.2)	5 (6.5)	0 (0.0)	0 (0.0)
Other	18 (11.5)	14 (18.2)	2 (3.3)	2 (10.5)
Schwannoma	32 (20.5)	11 (14.3)	21 (35.0)	0 (0.0)
Meningioma	25 (16.0)	0 (0.0)	25 (41.7)	0 (0.0)
Ependymoma	22 (14.1)	0 (0.0)	10 (16.7)	12 (20.0%)
Glioma	4 (2.6)	0 (0.0)	0 (0.0)	4 (21.1)
Sarcoma	3 (1.9)	3 (3.9)	0 (0.0)	0 (0.0)
Leiomyosarcoma	1 (0.6)	1 (1.3)	0 (0.0)	0 (0.0)
Round cell liposarcoma	1 (0.6)	1 (1.3)	0 (0.0)	0 (0.0)
Undifferentiated high‐grade spindle cell sarcoma lacking line differentiation	1 (0.7)	1 (1.3)	0 (0.0)	0 (0.0)
Other	17 (10.9)	16 (20.8)	1 (1.7)	0 (0.0)
ACCI (median, IQR)	2 (3)	3 (5)	1 (2)	2 (2)
Localization (*n*, %)				
Cervical	35 (22.4)	14 (18.2)	12 (20.0)	9 (47.4)
Cervicothoracic	7 (4.5)	2 (2.6)	1 (1.7)	4 (21.1)
Thoracic	62 (39.7)	39 (50.6)	20 (33.3)	3 (15.8)
Thoracolumbar	6 (3.8)	2 (2.6)	3 (5.0)	1 (5.3)
Lumbar	34 (21.8)	16 (20.8)	16 (26.7)	2 (10.5)
Lumbosacral	4 (2.6)	0 (0.0)	4 (6.7)	0 (0.0)
Sacral	8 (5.1)	4 (5.2)	4 (6.7)	0 (0.0)
Intra−/extradural (*n*, %)				
Extradural	77 (49.4)			
Intradural	79 (50.6)			
Intramedullary	−19 (12.2)			

*Note:* “Cervicothoracic”, “thoracolumbar”, and “lumbosacral” indicate tumor involvement spanning both respective spinal regions.

Abbreviations: ACCI, Age‐adjusted Charlson Comorbidity Index; IQR, interquartile range.

### 3.2 Tumor Localization and Surgical Parameters

An overview of the surgical approach and complication profile is given in Table 2: In the overall cohort, the predilection site was the thoracic spine (*n* = 62, 39.7%), followed by cervical (*n* = 35, 22.4%) and lumbar (*n* = 34, 21.8%) manifestations. Thoracic involvement was especially pronounced in the metastases (*n* = 32/53, 60.4%) and meningioma cases (14/25, 56.0%). Schwannomas and ependymomas were expectedly most common in the lumbar spine (10/32, 31.2% and 11/22, 50.0%, respectively). Intra‐ and extradural tumor manifestations were almost equal within this cohort (intradural *n* = 79, 50.6%, extradural: *n* = 77, 49.4%). Among the intradural lesions, 19 were intramedullary, including 12 ependymomas, 4 gliomas, and 3 metastatic cases. Among the latter, the primary tumors were breast carcinoma, melanoma, and parotid carcinoma (one case each). The most common surgical strategy for tumor resection was laminectomy/laminoplasty (*n* = 94, 60.3%, broken down in Table 2). Instrumentation procedures had to be applied in 34 (21.8) of the cases. Circumferential stabilization, mostly in the context of dorsal instrumentation and subsequent vertebral body replacement, was performed in 6 cases (3.8%). Cement augmentation for pedicle screws was only used in two cases within this cohort. Intraoperative neuromonitoring (including motor‐ and somatosensory evoked potentials) was used in the majority of intradural tumor surgeries including all intramedullary tumors (overall: 64/77 intradural cases, 83%). Estimated blood loss differed between tumor subgroups, with the highest values observed in extradural tumors (mean 521.4 mL), followed by intramedullary (407.9 mL) and intradural extramedullary tumors (243.5 mL).

**TABLE 2:** Surgical parameters and complication profiles.

Localization	Overall cohort	Extradural	Intradural extramedullary	Intradural intramedullary
	*n* = 156	*n* = 77	*n* = 60	*n* = 19
Duration of surgery (mean, SD)	199.9 (102.1)	187.3 (96.9)	194.6 (89.4)	266.3 (135.8)
Blood loss in mL (mean, SD)	400.7 (466.7)	521.4 (569.0)	243.5 (235.2)	407.9 (427.3)
Stay at the intensive care unit, *n* (%)	10 (6.4)	6 (7.8)	2 (3.3)	2 (10.5)
Urgency				
Elective	120 (76.9)	46 (59.7)	54 (93.3)	18 (94.7)
Emergency	36 (23.1)	31 (40.3)	4 (6.7)	1 (5.3)
Surgical approach				
Laminectomy	51 (32.7)	22 (28.6)	24 (40.0)	5 (26.3)
Laminoplasty	43 (27.6)	3 (3.9)	27 (45.0)	13 (68.4)
Hemilaminectomy	16 (10.3)	9 (11.7)	7 (11.7)	0 (0.0)
Interlaminar fenestration	4 (2.6)	2 (2.6)	2 (3.3)	0 (0.0)
Laminectomy + instrumentation	34 (21.8)	34 (44.2)	0 (0.0)	0 (0.0)
Other	8 (5.1)	7 (9.1)	0 (0.0)	1 (5.3)
Instrumentation appraoch				
Anterior	0 (0.0)	0 (0.0)	0 (0.0)	0 (0.0)
Posterior	28 (17.9)	28 (36.4)	0 (0.0)	0 (0.0)
Combined anterior–posterior approach	6 (3.8)	6 (7.8)	0 (0.0)	0 (0.0)
Cement‐augmented pedicle screws	2 (1.3)	2 (2.6)	0 (0.0)	0 (0.0)
Complications				
Yes	26 (16.7)	10 (13.0)	8 (13.3)	8 (42.1)
No	130 (83.3)	67 (87.0)	52 (86.7)	11 (57.9)
Clavien Dindo‐Grade				
I	8 (5.1)	0 (0.0)	4 (6.7)	4 (21.1)
II	7 (4.5)	4 (5.2)	1 (1.7)	2 (10.5)
III	8 (5.1)	4 (5.2)	2 (3.3)	2 (10.5)
V	3 (1.9)	2 (2.6)	1 (1.7)	0 (0.0)
Reoperation (Clavien‐Dindo ≥ 3)				
Yes	8 (5.1)	4 (5.2)	2 (3.3)	2 (10.5)
No	3 (1.9)	2 (2.6)	1 (1.7)	0 (0.0)
Complication type				
Surgical	23 (14.7%)	6 (7.8%)	8 (13.3%)	9 (47.4%)
Impaired wound healing	4 (2.6%)	3 (3.9%)	1 (1.7%)	0 (0.0%)
CSF fistula	2 (1.3%)	0 (0.0%)	1 (1.7%)	1 (5.3%)
New neurological deficit	13 (8.3%)	0 (0.0%)	6 (10.0%)	7 (36.8)
Postoperative hemorrhage	3 (1.9%)	3 (3.9%)	0 (0.0%)	0 (0.0%)
Implant malposition/displacement	0 (0.0)	0 (0.0%)	0 (0.0%)	0 (0.0%)
Other	1 (0.6%)	0 (0.0%)	0 (0.0%)	1 (5.3%)
Non‐surgical	6 (3.8%)	5 (6.5%)	1 (1.7%)	0.00%
Urinary tract infection	3 (1.9%)	3 (3.9)	0 (0.0%)	0 (0.0%)
Cardiac decompensation	1 (0.6)	1 (1.3%)	0 (0.0%)	0 (0.0%)
Mesenteric ischemia	1 (0.6)	0 (0.0%)	1 (0.0%)	0 (0.0%)
Pneumonia	1 (0.6)	1 (1.3%)	0 (0.0%)	0 (0.0%)

Abbreviations: CSF, cerebrospinal fluid; SD, standard deviation.

### 3.3 AEs

Within the overall cohort, complications were documented in 26 cases (16.7%). Out of these, 23 (88.5%) were surgical and 6 non‐surgical (23.1%). In three patients, both a surgical and nonsurgical adverse event occurred. Wound healing disorders occurred in 4 cases (2.6%)—purulence was only seen in one case (0.6%). CSF fistulas were observed in 2 cases (1.3%), postoperative hemorrhage in 3 cases (1.9%) and new postoperative deficits in 13 cases (8.3%). Within the reported wound healing disorders, one occurred during the initial hospital stay, while three patients required readmission after discharge. Three patients required surgical revision. One case represented a purulent wound infection requiring prolonged antibiotic treatment, whereas two additional cases were superficial wound healing disturbances treated surgically with prophylactic antibiotics until intraoperative cultures returned negative. The fourth case did not require reoperation. The patient presenting with a purulent wound infection was an 81‐year‐old man (ACCI = 10) who underwent a laminectomy at C5‐7 for decompression of the spinal canal due to spinal metastasis from prostate cancer. Duration of surgery was 80 min with a blood loss of 80 mL. The other two patients who underwent surgery for wound healing disorders were a 63‐year‐old patient with sarcoma (ACCI = 4), who underwent laminectomy with dorsal fixation of the thoracic spine, and an 80‐year‐old patient with meningioma (ACCI = 6), who underwent tumor resection via laminectomy at the level of T9/10. Duration of surgery were 280 min and 125 min respectively.

Stratified analysis revealed marked differences in complication profiles between tumor subgroups. Intramedullary tumors showed the highest overall complication rate (47.4%), predominantly driven by new neurological deficits (36.8%). In contrast, extradural tumors were more frequently associated with wound‐related complications and postoperative hemorrhage. Intradural extramedullary tumors demonstrated an intermediate risk profile.

Serious AEs (Clavien‐Dindo grade ≥ 3) were documented in 11 cases (7.1%); reoperation was performed in 8 cases (5.1%): The main indications included CSF fistula (*n* = 2), wound complications (*n* = 3), and postoperative epidural hemorrhage (*n* = 2). In addition, one reoperation was performed due to dislocation of a cranial bone flap related to head fixation in the Concorde position during cervical spine surgery. CSF fistulas occurred in patients with intradural tumors (one intramedullary metastasis and one myxopapillary ependymoma). Wound revisions included two cases of aseptic wound dehiscence and one surgical site infection (meningioma, leiomyosarcoma, and metastasis, respectively). Epidural hemorrhages occurred in patients with chondrosarcoma and prostate cancer metastases. Death within 30 days postoperatively was documented in 3 cases (Clavien‐Dindo V): One patient (86 years) with a hemorrhagic prostate cancer metastasis involving the thoracic spine (T4‐T9) and associated spinal cord compression underwent surgical decompression by hemilaminectomy at levels T4‐T9. Postoperatively, the patient developed a fulminant hemorrhage extending throughout the spinal canal to the craniocervical junction, resulting in tetraplegia. The patient declined revision surgery and opted for palliative management. A second patient (75 years) with spinal prostate cancer metastases underwent laminectomy from T6 to T11 and subsequently suffered cardiac arrest. The third patient (85 years), also diagnosed with prostate cancer, presented with an intramedullary metastasis at T10‐T11 and underwent partial tumor resection via laminectomy. Postoperatively, the patient developed peripheral cyanosis and clinical deterioration, ultimately progressing to asystole. Mesenteric ischemia related to tumor progression was suspected post hoc as the underlying cause. Accordingly, all fatal postoperative complications occurred in patients with prostate cancer metastases. The respective ACCI scores in these patients were 11, 12, and 10. No comparable accumulation of severe or fatal complications was identified among patients with other primary tumor entities.

### 3.4 Predictors for Surgical, Overall and Serious AEs

We assessed potential predictors of surgical complications, overall complications as well as serious complications (Table 3). Comorbidity burden was significantly associated with overall complications (*p* = 0.006) as well as serious complications (*p* = 0.001). There was a 39% increase in risk for serious AEs per ACCI‐point. Longer durations of surgery increased the risk for surgical as well as overall complications by 0.5% and 0.4% per minute (*p* = 0.022 and *p* = 0.046 respectively). However, there was no significant association with serious complications. Patients treated in the intensive care unit exhibited a significantly increased risk of overall, surgical, and serious complications (*p* < 0.001). Intradural tumor manifestation was a predictor for surgical complications—mainly worsening of neurological status (*p* = 0.031, OR = 3.005).

**TABLE 3:** Binary logistic regression analysis of predictors for postoperative complications.

	Complications (in general) OR, 95% CI (*p*)	Surgical complications OR, 95% CI (*p*)	Serious complications (Clavien‐Dindo ≥ 3) OR, 95% CI (*p*)
Age	(0.082)	(0.139)	1.06, 1.01–1.12 (**0.026**)
Sex	(0.119)	(0.241)	(0.891)
Level	(0.193)	(0.178)	(0.855)
Histology^a^	(0.999)	(0.993)	(0.986)
Metastasis^a^ (yes)	(0.155)	(0.799)	3.77, 1.05–13.51 (**0.042**)
Intradural localization	(0.227)	3.005, 1.108–8.150 **(0.031)**	0.722
ACCI	1.20, 1.05–1.36 (**0.006**)	(0.096)	1.39, 1.15–1.67 (< **0.001**)
Duration of surgery (min)	1.004, 1.000–1.007 (**0.046**)	1.005, 1.001–1.008 (**0.022**)	(0.310)
Estimated blood loss	(0.299)	(0.884)	(0.892)
Emergency surgery (yes)	(0.311)	(0.615)	4.6, 1.3–16.1 (**0.017**)
Intensive care (yes)	(15.6, 3.7–65.6) (< **0.001**)	12.2, 3.1–47.8 (< **0.001**)	(13.2, 3.0–57.9) (< **0.001**)
Surgery with instrumentation	(0.390)	(0.137)	(0.311)

Abbreviations: ACCI, age‐adjusted Charlson comorbidity index; CI, confidence interval; IQR, Interquartile range; min, minutes; OR, Odd's ratio (only given for significant associations).

^a^ “Histology” refers to an analysis where all histologies were tested without further classification; “Metastasis” refers to an analysis where histology was dichotomously divided: metastasis versus other entities. Bold values indicate statistical significance (*p* < 0.05).

### 3.5 New Neurological Deficit

New postoperative deficits were the most common surgical complication within this cohort. However, reoperation was only performed in one particular case: The patient underwent resection of an intramedullary melanoma metastasis at the C4 level. Due to cranial fixation in the prone position, a previous craniotomy became dislodged and required refixation. New neurological deficits were mainly seen in ependymomas (6/13, 46.2) and metastases (4/13, 30.8). All patients with new neurological deficits showed intradural tumor manifestations (Fisher's exact test, *p* < 0.001). In accordance with these histological patterns, 11 of the 13 cases (84.6%) with new deficits were operated via laminoplasty or laminectomy, hence reflecting the surgical access to the spinal canal. None of the instrumentation cases showed new neurological worsening.

## 4 Discussion

### 4.1 Main Results

In this cohort of 156 spinal tumor surgeries, we observed a moderate overall complication burden (16.7%), with a clinically significant rate of serious complications (Clavien–Dindo ≥ 3; 7.1%), reoperations (5.1%), and mortality (1.9%). Complications were predominantly surgery‐related (14.7%), with new postoperative neurological deficits being the most frequent single AE (8.3%). In multivariable analysis, comorbidity burden (ACCI) and intensive care treatment were the most consistent predictors of both overall and serious complications. Additionally, older age and emergency surgery were specifically associated with serious AEs, while operative duration showed a modest association with overall complications.

### 4.2 Heterogeneity and Stratified Complication Profiles

The stratified analysis underscores the structured heterogeneity of spinal tumor populations and its impact on adverse event profiles. Intramedullary tumors exhibited the highest complication rates, primarily driven by neurological deficits, reflecting the inherent risks of direct spinal cord manipulation. Sandalcioglu et al. reported postoperative neurological deterioration in 34.6% of patients within a cohort of 78 intramedullary spinal cord tumors, despite the use of intraoperative monitoring [15]. They identified preoperative status as the strongest predictor of functional outcome [15], which is consistent with our findings, where the ACCI also emerged as an important predictor. In a mixed cohort of intra‐ and extramedullary intradural tumors, postoperative neurological deterioration occurred in 18% of intramedullary tumors compared to 4% of extramedullary tumors [16]. In contrast, extradural tumors—largely metastatic—were associated with overall higher comorbidity burden, more frequent emergency procedures, and a predominance of wound‐related and hemorrhagic complications. In addition, estimated blood loss differed between subgroups, with higher values in extradural tumors, likely reflecting the increased need for instrumentation and the vascularity of metastatic lesions. However, blood loss was not significantly associated with overall, surgical or serious complications. Intradural extramedullary tumors showed a more favorable risk profile, consistent with their predominantly benign nature. However, due to dural opening, they inherently carry a risk of CSF leakage [16]. These findings highlight that adverse event patterns are closely linked to tumor biology and surgical context, supporting the importance of stratified analyses in future spinal oncology studies.

### 4.3 Comparison With Registry Benchmarks and the Impact of Outcome Definitions

Our findings align with large‐scale registry data. An analysis of NSQIP data (2011–2014) reported major complications in 14.4%, reoperations in 5.3%, and a 30‐day mortality of 3.3%, noting that a substantial proportion of complications occur post‐discharge [11]. More recently, Kazim et al. [17] (NSQIP 2015–2019) reported a lower 30‐day mortality of ~1.6% and highlighted the value of frailty measures for risk stratification. While our mortality (1.9%) and reoperation rates (5.1%) are comparable to these benchmarks, interpretation depends heavily on follow‐up duration and the granularity of AE reporting. A critical limitation of general surgical datasets is their focus on systemic outcomes (e.g., readmission, mortality) at the expense of spine‐specific endpoints like neurological deficits [18]. This is a crucial distinction, as neurological deterioration is central to clinical decision‐making and represents a major component of the “true” AE burden, even if it does not trigger a reoperation. In our cohort, new neurological deficits were the most common AE (8.3%)—a rate comparable to previous studies [19]—reinforcing neurological preservation as the primary safety endpoint in spinal oncology.

### 4.4 CSF‐Fistula and Wound Complications

We observed CSF fistulas in 1.3% of cases and impaired wound healing in 2.6%. Reported CSF leak rates vary widely in the literature, often depending on whether incidental or only clinically significant leaks are counted; Barber et al. summarized an incidence range of 0%–28.6% for leaks requiring intervention, with a mean of approximately 6.6% [20]. Our lower rate likely reflects a combination of factors: (i) a mixed case‐mix of intradural and extradural pathologies, (ii) a strict definition focused on clinically relevant leaks, and (iii) the potential under‐capture of delayed leaks presenting after discharge. The latter is particularly relevant given evidence that many spinal tumor complications manifest only after hospital discharge [11]. Regarding wound complications, our rate was lower than many reports from metastatic spine surgery, where surgical site infection rates around 6.5% have been reported in systematic review data [21]. Vargas et al. report an incidence of 11.2%–14.5% depending on preoperative radiation exposure [22]. Several factors may explain the lower wound complication rates in our cohort: First, our cohort included a substantial proportion of benign intradural lesions and comparatively fewer patients exposed to classical wound risk amplifiers common in purely metastatic populations, such as prior radiotherapy or systemic therapy [23, 24]. Accordingly, instrumentation was also used less often than in metastatic cohorts [25]. Second, an important center‐specific factor may be our routine preference for percutaneous stabilization techniques in metastatic cases, where only the metastatic levels are openly decompressed. Minimally invasive and percutaneous approaches have been associated with lower overall and wound complications compared with open surgery, likely due to reduced soft tissue trauma, smaller dead space, and lower bacterial burden [26, 27, 28]. Finally, our comparatively low wound complications rate may also partly relate to the study's 30‐day observation window, as delayed wound events may occur beyond this period and therefore may not be fully captured in our analysis.

### 4.5 Predictors of Complications

The most consistent risk signal in our analysis was the ACCI and intensive care unit stay, associated with increased odds of both overall and serious complications. This aligns strongly with metastasis‐specific evidence: Arrigo et al. identified the ACCI as the strongest predictor of 30‐day complications following surgery for spinal metastases, with an odds ratio exceeding 5 in patients with an ACCI ≥ 2 [29]. Beyond comorbidity counts, modern registry work highlights the incremental predictive value of frailty [17]. Our finding that age was associated with serious complications is consistent with the broader concept of declining physiological reserve. This provides a clear discussion trajectory: short‐term AEs after spinal tumor surgery may be best understood as being driven primarily by host factors (comorbidity/frailty) and clinical context (urgency), rather than tumor entity in isolation.

### 4.6 The Role of Urgency

In our cohort, emergency surgery was associated with markedly increased odds of serious complications. This aligns with evidence that “high‐acuity” oncologic spine surgery populations exhibit substantial morbidity: Dea et al. prospectively analyzed emergency oncological spine surgery using SAVES‐V2 (Spine Adverse Events Severity System, version 2) forms and reported a very high adverse event burden and notable in‐hospital mortality, emphasizing the fragility of this subgroup and the importance of preventive strategies [30]. Although our cohort included benign pathologies, the association suggests that emergency status serves as a composite marker for preoperative neurological compromise, systemic vulnerability, and a lack of time for physiological optimization. Similarly, while “intensive care stay” was a strong statistical predictor, it must be interpreted with caution; intensive care unit admission may represent planned monitoring for high‐risk cases or, conversely, a rescue pathway following a complication (reverse causality).

### 4.7 Histological and Anatomical Considerations

In our logistic regression analysis, intradural tumor manifestations were significantly associated with surgical complications. Given that the majority of surgical complications consisted of neurological deficits, this association is plausible in the context of spinal cord manipulation and CSF leakage resulting from durotomy. Spinal level was not associated with overall or serious complications. Contrarily, previous studies have identified specific anatomical high‐risk constellations: particularly anterior and lateral thoracic IDEM (intradural extramedullary) tumors were associated with increased surgery‐related complications, likely due to regional anatomical and vascular constraints [19, 31]. When analyzing tumor histologies altogether, we found no significant association with complications. However, when dichotomizing the dataset (metastases vs. other entities), there was a significant risk burden of metastases for serious AEs. Metastatic disease has been reported as a predictor of postoperative complications in other mixed spinal surgery cohorts as well [32]. Within this cohort, death within 30 days was exclusively observed in patients with prostate cancer metastases. However, this apparent clustering of fatal AEs warrants cautious interpretation, as these patients showed markedly high ACCI scores (10, 11, and 12) and older age (75, 85 and 86 years). This suggests that the observed outcomes may primarily reflect advanced systemic disease burden and limited physiological reserve rather than tumor‐specific risk. Not only was the ACCI a significant predictor of overall and serious complications within our cohort, but it was also previously suggested to be a significant independent predictor of prognosis in elderly cancer patients [33]. Specifically, a meta‐analysis examining the association between the CCI and survival outcomes in prostate cancer patients reported a pooled hazard ratio of 3.95 for other‐cause mortality in patients with a CCI score ≥ 3 [34].

### 4.8 Instrumentation and Operative Duration

Instrumentation was not associated with higher complication risk in our cohort. In metastatic populations, instrumentation failure is reported at relatively low rates compared with infection, and reoperations frequently occur for wound complications rather than hardware issues [21]. Our findings support the interpretation that stabilization, when indicated, should not be assumed to be the primary driver of AEs. Operative duration showed a small association with overall complications, plausibly reflecting surgical complexity and exposure time rather than a direct causal mechanism.

### 4.9 Neurological Deficits and Intradural Tumor Surgery

Despite the frequent use of intraoperative neuromonitoring, new postoperative neurological deficit was the most frequent surgical AE in our cohort (8.3%) and occurred exclusively in intradural tumors. This finding is consistent with intradural tumor series identifying neurological deterioration as a leading complication category [19]. Notably, reoperation was rarely required among patients with new deficits, suggesting that most cases reflected intrinsic spinal cord or nerve root injury/ischemia/edema rather than a readily reversible compressive etiology.

The clustering of deficits in ependymomas and (rare) intradural metastases likely reflects the technical and biological challenges of intramedullary or cord‐adjacent disease. Myelotomy and dissection along functional white‐matter tracts, manipulation of radiculomedullary vessels, and postoperative cord swelling can translate into early neurological decline even after adequate decompression. Because AEs were captured within 30 days, we cannot distinguish transient postoperative worsening from permanent deficits, which is a clinically important nuance for patient counseling and for comparisons across cohorts.

Two procedural observations warrant cautious interpretation. First, the predominance of laminectomy/laminoplasty among deficit cases likely mirrors the access required for intradural lesions rather than an AE of the approach itself. Second, the absence of new deficits in instrumented cases should not be interpreted as a protective effect of stabilization; instead, it likely reflects confounding by indication, as instrumentation was primarily used for extradural/instability indications where neurological risk is less dominated by intradural cord manipulation. Prior work suggests that specific anatomical subgroups, particularly anterior or upper thoracic intradural tumors, may carry higher neurological risk even when global level effects are not detected in mixed cohorts [31].

### 4.10 Implications for Practice and Future Reporting

Our data support three pragmatic implications. First, structured risk stratification using comorbidity or frailty metrics should be integrated into preoperative counseling and care pathways [17]. Second, the strong link between serious complications and emergency surgery highlights the value of early referral and multidisciplinary triage to facilitate elective management whenever possible [30]. Third, given that neurological deficits were the most frequent complication, safety strategies must prioritize neuroprotection and vigilant postoperative neurological surveillance. Finally, the variability in reported outcomes emphasizes the need for standardized definitions. While SAVES‐V2 offers high reliability for spine‐specific events [35], the Clavien–Dindo system—though useful for broad comparability—may underestimate severe events like neurological deficits that do not strictly require “intervention,” a limitation previously noted in neurosurgical validation studies [36]. Nevertheless, we selected Clavien–Dindo as it provides a standardized, therapy‐oriented framework that facilitates comparison across surgical disciplines. In addition, its routine use across all neurosurgical subspecialties at our institution supports consistent and uniform adverse event reporting. These frameworks should therefore be considered complementary rather than mutually exclusive.

### 4.11 Limitations

The principal strength of this study is the use of a prospective database with direct post‐discharge documentation for each patient. Nonetheless, several limitations warrant consideration. First, our analysis is limited to early POPAE within 30 days. While this timeframe captures complications most relevant to perioperative management and is widely used for benchmarking, it does not account for delayed events such as instrumentation failure or late wound complications. Therefore, our findings should be interpreted as reflecting early surgical risk rather than the full spectrum of postoperative morbidity. Future studies with extended follow‐up are warranted to complement these findings [9, 10, 11]. Second, the heterogeneity of tumor pathologies and surgical objectives may dilute entity‐ or location‐specific effects, while the relatively small number of serious AEs constrains the precision of subgroup risk estimates. In addition, certain variables, such as intensive care unit admission, likely capture both preoperative planning and postoperative clinical course, thereby complicating causal interpretation. Finally, the single‐center design limits generalizability; a multicenter approach would enhance the external validity of these findings.

## 5 Conclusion

In a mixed spinal tumor surgery cohort, we found a moderate overall complication burden but a clinically meaningful rate of serious AEs and reoperations. The complication profile was dominated by surgery‐related events, particularly new neurological deficits. Risk was driven primarily by patient vulnerability (ACCI/Intesive care unit stay), age, and emergency status, reinforcing the importance of preoperative risk stratification and pathway optimization to reduce emergency presentations and improve perioperative safety.

## Author Contributions

**Meltem Ivren:** writing – original draft, writing – review and editing, formal analysis, data curation, conceptualization, investigation, methodology. **Philip Dao Trong:** methodology, writing – review and editing, supervision. **Maximilian Klass:** investigation, software, data curation. **Martin Dugas:** validation, supervision. **Sebastian Ille:** validation, supervision. **Sandro M. Krieg:** validation, supervision. **Pavlina Lenga:** conceptualization, methodology, validation, writing – review and editing, supervision, project administration.

## Ethics Statement

The local ethics committee approved this study (approval number S‐425/2022). As POPAE forms are completed for every patient treated at our center as part of our standard clinical procedure, we did not specifically gather informed consent.

## Conflicts of Interest

The authors declare no conflicts of interest.

## Data Availability

The data that support the findings of this study are available from the corresponding author upon reasonable request.
